# Site-Specific Secretome Map Evidences VSMC-Related Markers of Coronary Atherosclerosis Grade and Extent in the Hypercholesterolemic Swine

**DOI:** 10.1155/2015/465242

**Published:** 2015-08-25

**Authors:** Silvia Rocchiccioli, Antonella Cecchettini, Nadia Ucciferri, Marianna Terreni, Federica Viglione, Maria Giovanna Trivella, Lorenzo Citti, Oberdan Parodi, Gualtiero Pelosi

**Affiliations:** ^1^National Research Council, Institute of Clinical Physiology, Via Moruzzi, 56124 Pisa, Italy; ^2^Department of Clinical and Experimental Medicine, University of Pisa, Via Volta, 56126 Pisa, Italy

## Abstract

A major drawback in coronary atherosclerosis (ATS) research is the difficulty of investigating early phase of plaque growth and related features in the clinical context. In this study, secreted proteins from atherosclerotic coronary arteries in a hypercholesterolemic swine model were characterized by a proteomics approach and their expression was correlated to site-specific ATS stage and extent. A wide coronary artery map of secreted proteins has been obtained in high fat (HF) diet induced ATS swine model and a significantly different expression of many proteins related to vascular smooth muscle cell (VSMC) activation/migration has been identified. Significant associations with ATS stage of HF coronary lesions were found for several VSMC-derived proteins and validated for chitinase 3 like protein 1 (CHI3L1) by tissue immunoexpression. A direct correlation (*R* ^2^ = 0.85) was evidenced with intima to media thickness ratio values and ELISA confirmed the higher blood concentrations of CHI3L1 in HF cases. These findings confirmed the pivotal role of VSMCs in coronary plaque development and demonstrated a strong site-specific relation between VSMC-secreted CHI3L1 and lesion grade, suggesting that this protein could be proposed as a useful biomarker for diagnosing and staging of atherosclerotic lesions in coronary artery disease.

## 1. Introduction

Coronary atherosclerosis (ATS) is the underlying pathology of major acute events associated with thrombotic obstruction of vessel lumen and a leading cause of morbidity and mortality in Western countries [1, 2].

Identification of molecular factors associated with coronary atherogenesis (i.e., coronary plaque formation and early progression) has relevant implications for diagnosis, prevention, and treatment of overt coronary artery disease (CAD), especially when significant associations emerge between these factors and the ATS grade, lesion extent, and progression.

Within this context, proteome profiling represents an interesting approach to disclose a broad spectrum of cellular proteins and peptides and to propose putative biomarkers [3]. A major obstacle in investigating coronary ATS initiation and progression is related to the advanced stage of human atherosclerotic samples collected at surgery and the consequent difficulties in digging putative biomarkers in a complex tissue proteome, characterized by a wide dynamic range of concentration. The study of secreted factors in atherosclerosis research could override the limits of a plaque proteome [4] evidencing hidden actors and representing a valuable source of noninvasive markers detectable in blood.

In order to reduce sample complexity in proteome studies and to obtain human tissue representatives of the early stages of plaque formation and progression, de la Cuesta et al. have recently [5] analyzed the medial layer of atherosclerotic coronary arteries by 2D-DIGE, highlighting a differential expression of several cytoskeleton proteins of atheroma-adjacent VSMCs as compared to preatherosclerotic cells, and hypothesized a key role in plaque formation by activated VSMCs.

The same authors [6] have previously used a label-free liquid chromatography approach coupled with mass spectrometry (LC-MS/MS) analyses to compare the secretome data from human atherosclerotic coronary arteries with preatherosclerotic coronaries and intact mammaries (collected at autopsy) and identified four VSMC-related proteins, which were downreleased in the pathological samples.

Both proteomics approaches highlighted the key role of arterial VSMCs in atherosclerotic plaque initiation and growth. Moreover, this role was related to the well-known VSMC phenotypic heterogeneity, which accounts for various cell activities such as proliferation, migration, and synthesis/degradation of extracellular matrix (ECM) components [7]. Indeed, according to the classical theory of atherogenesis, after inflammatory cells and mediators initiate the process by foam cell formation, the overexpression of ECM proteoglycans (ECM-PGs), synthetized by activated VSMCs, would enhance subendothelial lipoprotein trapping while VSMC proliferation contributed to plaque growth and to its evolution towards stability [8].

A clear association of the full spectrum of lesion stages in coronary atherogenesis with known or novel molecular factors of pathology progression is currently missing for several reasons: (i) the inherent difficulty to investigate the early stage of ATS [9] and to identify low-abundant cell-secreted proteins; (ii) the limited number of studies which have focused on protein expression in the intima layer of early coronary lesions in humans and the even lower number of those that have compared preatherosclerotic with advanced coronary lesions [10–13]; (iii) the difficulty of assessing which of the observed coronary preatherosclerotic lesions, such as diffuse intimal thickening and fatty streaks, are prone to progress into advanced lesions and which will remain stable over decades, a condition that limits the identification of informative markers of evolving lesions [9].

All these limitations in studying plaque onset and formation have encouraged the use of animal models, among which swine is considered the closest to human. [14]. Although genomics and transcriptomics atherogenesis-related changes are reported in mouse and swine models [15, 16], a wide proteomics profiling of porcine coronary arteries, to identify molecular factors and protein pathways involved in plaque initiation and early formation, has not yet been described.

Aims of the present study are (i) to identify a molecular map of proteins secreted by intact and atherosclerotic coronary arteries of pigs fed on standard (CTRL) and hypercholesterolemic (HF) diet, respectively, using a hypothesis-free approach; (ii) to compare protein expressions of CTRL and HF samples and to evidence differentially secreted proteins that could be related to CAD onset and progression; and (iii) to associate the most relevant differentially expressed proteins with histomorphometrically characterized atherosclerotic lesions and to identify their cellular localization by immunohistochemistry.

## 2. Methods

### 2.1. Experimental Protocol

#### 2.1.1. Animals and Diet

The protocol was applied to 12 male domestic pigs of 8 to 12 weeks of age. Animals were allocated into two groups: controls fed on standard chow (CTRL, *n* = 6) and animals fed on high fat cholesterol-enriched diet (HF, *n* = 6) for 4 months (119 days). Mean baseline body weight in the two groups was not significantly different and raised to 42 ± 7 Kg and 49 ± 7 Kg in CTRL and HF, respectively, at the end of diet period (mean values ± SD, NS). High fat diet, as compared to standard one, was supplemented with 20% lard and 4% cholesterol (4450 Kcal/kg with 54.6% of total energy provided by fat). The plasma lipid profile was evaluated: plasma triglycerides (TGs), high-density lipoprotein (HDL), low-density lipoprotein (LDL), and total cholesterol (TC) content were measured by enzymatic colorimetric reactions using commercial kits (Synchron CX9 Pro, Beckman Coulter Inc., USA). Values (mg/dL) in the CTRL group (*N* = 6, mean ± SD) were as follows: TGs: 29.5 ± 19.3; TC: 58.3 ± 4.9; HDL: 25.5 ± 4.4; LDL: 27 ± 5.5; TC/HDL ratio: 2.3 ± 0.2; values in HF group (*N* = 6, mean ± SD) were as follows: TGs: 65 ± 45; TC: 558 ± 134; HDL: 34 ± 14; LDL: 511.2 ± 133.6; TC/HDL ratio: 19 ± 7. LDL was calculated according to Friedewald et al. [17]. Apolipoprotein A1 was measured by rate nephelometry (BN-ProSpec, Siemens Healthcare Diagnostics, Italy): CTRL group (*N* = 6, mean ± SD): 22.8 ± 8.5 mg/dL; HF group (*N* = 6, mean ± SD): 54.7 ± 6.4 mg/dL. All values were significantly different between CTRL and HF group.

#### 2.1.2. Surgery

Anaesthesia was induced by intramuscular administration of 10 mg/kg of Zoletil and 0.05 mg/Kg of Atropine and maintained with gas (isofluorane, nitrous oxide, and oxygen) together with 5 mg/kg/h of Propofol intravenous infusion. Animals were mechanically ventilated (respiratory volume: 150 mL/Kg/min, respiratory rate: 15 cycles/min) and sacrificed by KCl i.v. injection under anaesthesia.

#### 2.1.3. Tissue Processing

The femoral artery (FA) and the proximal tract of the right coronary artery (RCA) were isolated and 25–30 mm long segments excised and quickly placed in serum-free medium for secreted protein collection. Thereafter the entire heart was immersed in 5% buffered formalin for tissue fixation (5–7 days) and subsequent coronary segmentation for histology and immunohistochemistry.

### 2.2. Secreted Protein Collection Procedure

Immediately after heart arrest, RCA and FA segments from CTRL and HF cases were processed according to literature and collected proteins were analysed by HPLC-MS/MS analysis [18]. Briefly, samples were incubated in 6-well plates in 2 mL of Eagle's Minimal Essential Medium (Sigma-Aldrich, USA) supplemented with Penicillin and Streptomycin, without Fetal Bovine Serum (FBS) and Phenol Red at 37°C in a humidified atmosphere of 5% CO_2_. After three hours, the medium was replaced. After 24 h, the culture medium was harvested, centrifuged at 300 ×g for 10 min. Samples were concentrated by centrifugal devices Amicon Ultra-3 (Millipore, Germany) following the manufacturer's recommendations.

### 2.3. Reduction, Alkylation, and Digestion of Proteins

Each secretome sample was processed, by preparing a solution of 1 *μ*g/*μ*L of proteins and 40 mM of ammonium hydrogen carbonate (Sigma-Aldrich, USA). Reduction was obtained by adding 5 mM dithiothreitol to each sample, with an incubation of 20 min at 80°C. Finally alkylation was obtained by adding 10 mM iodoacetamide and incubation for 30 min at 37°C. Digestion was performed incubating the samples overnight with 0.25 mg/mL of trypsin solution at 37°C (substrate/enzyme = 100/1).

### 2.4. LC-MS/MS Analysis and Data Processing

Chromatographic separation of digested peptides was performed using an Ultimate 3000 nano-HPLC system (LC Packings, DIONEX, USA) and peptides eluted from chromatographic C18 column were directly processed using TripleTOF 5600 mass spectrometer (ABSCIEX, Canada). For each secretome sample (CTRL *N* = 6, HF FA *N* = 6 and HF RCA *N* = 6), two technical replicates were injected.

The mass spectrometer was controlled by Analyst 1.6.1 software (AB SCIEX, Toronto, Canada). For positive ionization, ion source parameters were the following: spray voltage was 3 kV and source temperature 150°C with curtain gas set at 25, GS1 10, and GS2 0 psi nitrogen flow. For information dependent acquisition (IDA) analysis, survey scans were acquired in 250 ms and 25 product ion scans were collected if exceeding a threshold of 125 counts per second (counts/s). The total cycle time was fixed to 1.25 s. Four time bins were added for each scan at a pulser frequency value of 11 kHz through monitoring of the 40 GHz multichannel TDC detector with four-anode/channel detection. Dynamic exclusion was set to 1/2 of peak width (~8 s), and then the precursor was refreshed off the exclusion list.

MS/MS data were processed with ProteinPilot Software (ABSCIEX, Canada), using the Paragon and Pro Group Algorithms and SwissProt 2013 as protein database for* Sus scrofa*. The false discovery rate (FDR) analysis was performed using the integrated tools in ProteinPilot software and a confidence level of 95% was set to exclude false positive sequence identifications by analyzing the specificity and the quality of results with decoy database searching. Expression data for proteins and label-free comparative analysis were obtained using MarkerView software 1.2.1 (ABSCIEX, Canada).

### 2.5. Western Blot Analysis of Secretome Samples

The same secretome samples used for HPLC-MS analysis (CTRL, HF) were tested on a 10% SDS-PAGE; separated proteins transferred onto a nitrocellulose membrane (Amersham, GE Healthcare, USA) using a wet transfer system (Bio-Rad Laboratories, USA). Membranes were blocked with 3% BSA in TBST for 1 h at room temperature. Primary and secondary antibodies were diluted in 3% BSA in TBST. All primary antibodies were incubated overnight at 4°C. HRP-conjugated secondary antibodies (Santa Cruz Biotechnology, USA) were incubated for 1 h at room temperature.

The following antibodies were used: CATD (C20), goat polyclonal (Santa Cruz Biotechnology, USA), dilution 1 : 300, and CH3L1 goat polyclonal (R&D Systems), dilution 1 : 500.

Densitometric quantification of photographic films was performed using Quantity One 1-D Analysis Software (Bio-Rad Laboratories, USA). Photographic films were scanned and analysed by Quantity One 1-D Analysis Software (Bio-Rad Laboratories, USA).

### 2.6. Histology and Immunohistochemistry

After incubation for secretome analysis, all segments of RCA and FA were placed in 5% buffered formalin and processed for paraffin embedding: longitudinal 5 *μ*m thick consecutive sections were obtained by a rotary microtome (Microm HM 300, Bio-optica) and stained with Haematoxylin and Eosin (H&E) and Masson's trichrome stain in order to observe and morphometrically assess pathologic changes. The same procedure and analysis were carried out on 2 additional cross-sectioned segments of RCA adjacent to those sampled for secretome analysis (distal and proximal side) in each case; other coronary samples were harvested from left main (*n* = 2), left anterior descending (*n* = 10), and left circumflex arteries (*n* = 4) in each CTRL and HF case and processed for histology in order to morphometrically assess overall coronary ATS burden. A total of 20 to 30 consecutive serial sections were obtained from each segment for histologic staining and immunohistochemistry. For immunohistochemistry, sections were placed on positively charged slides, deparaffinized, rehydrated, and washed in distilled water. After incubation in H_2_O_2_ at room temperature, antigen retrieval was accomplished (citrate buffer pH = 6 in microwave for 10 min at 500 W) and then sections incubated with diluted normal blocking serum. The following primary antibodies were used: anti-*α*SM-actin (alpha smooth muscle actin, clone 1A4 ADB, Serotec) as a VSMC phenotype marker, anti-S100 A4 (rabbit polyclonal antibody diluted 1 : 200, Novus Biologicals) as a marker of synthetic VSMC phenotype [19, 20], and anti-CHI3L1 (goat polyclonal antibody diluted 1 : 40, R&D Systems) as a marker derived from the results of secretome analysis. They were applied overnight on the slides in a 4°C humid chamber. Following 30 min biotinylated secondary antibody and 30 min Vectastain Elite ABC reagent incubation in Peroxidase substrate solution (DAB), slides were counterstained with Mayer's Haematoxylin for 1 min and mounted (Neo-Entellan Merk). Omission of the primary antibody served as a negative control. Antibody binding is visible as brown or dark brown stain (DAB); negative cells are stained blue (Haematoxylin counterstain).

### 2.7. Morphometry and Quantitative Immunohistochemistry

Consecutive longitudinal and cross sections of each arterial sample were examined under a light microscope (Olympus BX43) at 4x to 40x original magnification and digitized by a video system (Olympus D20 camera) interfaced to Olympus Cell Sens Dimension software for image acquisition and analysis.

Arterial wall changes were analysed by two independent pathologists who attempted to relate them to the current histological grading according to American Heart Association (AHA) classification guidelines [8, 21].

The following morphometric indexes were calculated by mean value of 5 consecutive cross sections of each identified lesion at the site of its maximal extent: maximal intimal thickness (IT), maximal intimal to media thickness ratio (IMT ratio), cross-sectional lesion area (LA), and lesion to intact wall area ratio (LA ratio).

All RCA segments used for secretome analysis were morphometrically analysed; additional RCA, left main, left anterior descending, and left circumflex artery segments were also analysed to estimate overall coronary ATS burden from morphometry in each case of HF group. Results were correlated with those of corresponding coronary samples processed for secretome analysis. A total number of 134 coronary segments were morphometrically analysed (see Table S4 in Supplementary Material available online at http://dx.doi.org/10.1155/2015/465242).

Quantitative analysis of antibody staining of full lesion area was carried out by averaging microscopic measurements of 3 consecutive sections of each segment, digitized at 20x magnification under the same light source settings, and processed by semiautomatic color thresholding of Olympus Cell Sens Dimension software: positive-staining area was expressed as percentage of dark brown pixels of the entire lesion area and used as a comprehensive index of antibody binding.

Tissue codistribution and cell colocalization of different antibodies in the same region and cell type, respectively, were assessed by comparing them with positive tissue/cell in the corresponding microscopic field of adjacent consecutive sections.

### 2.8. ELISA of Plasma Samples

Dosage by double-antibody sandwich enzyme-linked immunosorbent assay (ELISA) was performed for CHI3L1. ELISA kit was used and reagents were prepared following the manufacturer's manual of Uscn Life Science Inc. All standards and plasma samples collected before surgery from the same animals (CTRL *N* = 6 and HF *N* = 6) were assayed in duplicate. The OD absorbance at 450 nm was read by a FLUOstar Omega microplate reader (BMG Labtechmicroplate).

### 2.9. Data Analysis

To evaluate differentially released proteins by CTRL, HF RCA, and HF FA segments (the latter for internal control), MarkerView 1.2 software was used and principal component analysis (PCA) was performed on mass spectrometric data of individually different biological samples to assess global differences between the groups (Figure S5 in Supplementary Material). Comparative analysis in MarkerView was performed, comparing MS area of the peptide eluted peaks with total area of the peptide peaks for proteins. Normalization was accomplished by LC-MS profiles (normalization based on total MS derived total ion-current area as an estimate of total protein content). Two technical replicates for biological sample were used and mean values were obtained for comparative analysis (Table S3 in Supplementary Material). Principal component analysis (PCA) was performed in order to identify groups within the dataset. The three groups (CTRL, HF FA, and HF RCA) were compared by *t*-test statistics: CTRL versus HF FA, CTRL versus HF RCA, and HF FA versus HF RCA samples.

Paired *t*-test was used as statistical parameter between the means of continuous variables to determine significant differences between the two categories of mass spectrometric data. Fold change > 2 and *P* value < 0.05 were considered significant to validate differences between categories.

Statistical analyses of other data were conducted using Origin 7.0 software (Origin Lab, USA). Data were expressed as the mean ± SD. Differences between the means of the 2 continuous variables were evaluated by Student's *t*-test and results were accepted when *t*-test > 95% (*P* value < 0.05). Paired *t*-test was used for quantitative immunohistochemistry. Ingenuity pathway analysis (IPA, http://www.ingenuity.com/products/pathways_analysis.html) was performed on a restricted number of VSMC-related proteins (*n* = 31), selected by their best correlation with at least one morphometric index of lesion extent. The subset of chosen proteins constituted of smooth muscle cell synthesized proteins and their role in cell migration and proliferation and in vascular disease was evidenced.

## 3. Results

### 3.1. Histology and Histomorphometry

Vessel segments used for secretome analysis and adjacent segments were histologically characterized. RCA and FA of CTRL group displayed an intact intima. Less than 2% of examined coronary segments showed small myointimal cushions at branching points as previously described in pigs on standard diet [22]. FA segments of HF group evidenced intact intima in 4 cases and Stary type I lesions in 2 cases.

Conversely, RCA from HF pigs showed atherosclerotic changes of the intima, ranging from lesions classifiable as Stary types I–III in 3 cases (preatheroma changes, pre-ATH) to those resembling types IV-V atheromas in the other 3 cases (atheroma stage, ATH).

**Figure 1:**
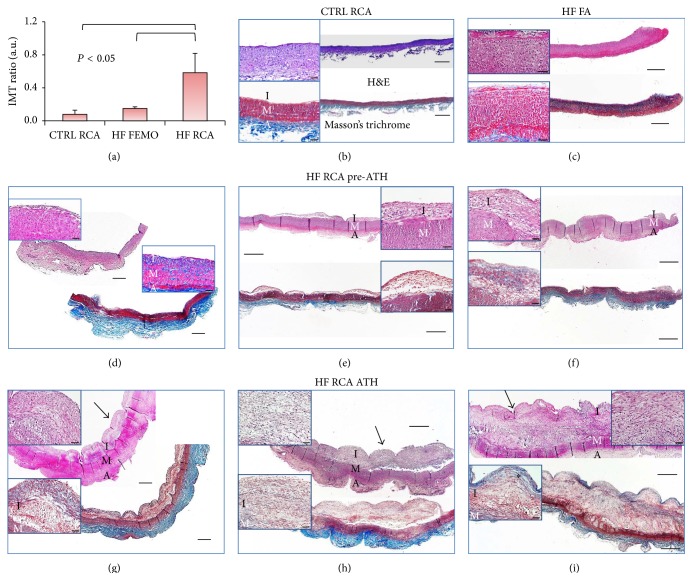
Morphometric results (panel (a)) and histologic characterization (panels (b)–(i)) of arterial segments used for secretome analysis. Panel (a): average values ± SD of intima to media ratio (IMT) at maximal intimal thickening site in CTRL RCA, HF FA, and HF RCA segments used for secretome analysis (*n* = 6 each). *P* < 0.05 HF RCA versus all other segments. Panels (b)–(i): representative photomicrographs of H&E and Masson's trichrome stained longitudinal sections of arterial segments: intact (b) or initial type I thickening (c) in typical CTRL RCA and HF FA segments, respectively; HF RCA segments showed preatherosclerotic lesions (HF RCA pre-ATH) classifiable as Stary type I lesion (d), type II fatty streak (e), and type III lesion (f), or atheromas (black arrows, HF RCA ATH segments) classifiable as type IV ((g) and (h)) and as type V fibroatheroma ((i), fibrous cap: asterisk). Low power micrographs, bar = 500 *μ*m, high power insets, bar = 50 *μ*m. I = intima, M = media, A = adventitia.

Histologic features and average values of IMT ratio of the segments processed for secretome analysis are shown in Figure 1.

Classification of lesions according to Stary staging of ATS and average histomorphometric results of RCA and FA segments are summarized in Table 1. Average values of IT, IMT ratio, LA, and LA ratio of RCA segments of HF group displaying only preatheroma (pre-ATH) changes and those with atheroma (ATH) are reported separately; the latter subgroup shows significantly higher values.

The average values of morphometric indexes according to lesion grade in all coronary segments sampled in CTRL and HF cases are reported in Table S4 in Supplementary Material.

### 3.2. Proteomics Profiling of Secreted Proteins and Comparative Analysis between CTRL and HF Data

**Figure 2:**
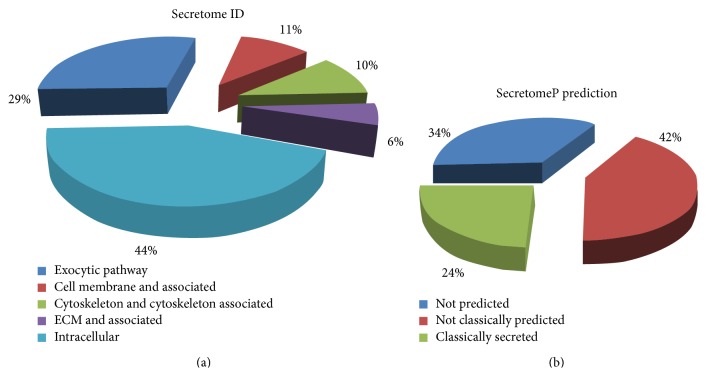
Pie charts of the total identified proteins from CTRL and HF. (a) Identified proteins are classified, based on Gene Ontology, according to their localization in intracellular or extracellular space. (b) Identified proteins are evaluated with SecretomeP software to compute their secretion potential. They were divided into classically secreted, not classically secreted, and not predicted.

224 proteins were identified across all samples with a Protein Score (Confidence) > 95% and local false discovery rate analysis < 1% as stringent criterion to avoid false positives (Table S1 in Supplemtary Material). Proteins were grouped using Gene Ontology (http://www.geneontology.org) and divided in exocytosis pathway related, cell membrane and associated, cytoskeleton and associated, intracellular, and ECM associated proteins (Figure 2(a)). The secretion potential of identified proteins was computed by submitting them to SecretomeP tool which uses specific databases to predict a classical secretion via endoplasmic reticulum (presence of signal peptide) or not classical secretion through the multivesicular bodies [23] (Figure 2(b)).

PCA unsupervised clusters (Figure S5 in Supplementary Material) were CTRL samples (*N* = 6), HF FA (*N* = 6), and HF RCA (*N* = 6), which resulted in two subgroups (pre-ATH and ATH, *N* = 3 each) histologically corresponding to different ATS stage, as reported in Table 1.

Paired *t*-test evidenced 17 differentially secreted proteins when all HF RCA samples were compared to CTRL. HF FA samples were used only to distinguish between diet-related and ATS-related tissue-secreted proteins. *t*-test between HF FA and CTRL groups, which was used to suggest diet-related factors, showed significant differential expression in glyceraldehyde 3 phosphate dehydrogenase, desmin, prelamin A/C, glutathione peroxidase 1, and apolipoproteins A I and A IV.

Differentially expressed proteins were grouped into cellular (*N* = 11) and extracellular matrix proteins (*N* = 6) (Table 2). The putative roles of each factor in atherogenesis and in disease-related cellular pathways, as suggested by the literature, are reported in Table 2.

Ten out of these proteins had already been reported in serum/plasma and listed in Human Protein Reference database (http://www.hprd.org) and 15 resulted as predicted to be secreted by SecretomeP software. Western blot analysis was applied to two identified markers to validate the expression of intact proteins by an antibody-based approach and observed by mass spectrometric analysis using expression of digested peptides. Additionally, by comparing pre-ATH (*N* = 3) and ATH (*N* = 3) segments (see Figure S2 in Supplementary Material), a statistical significant difference was observed only for CHI3L1 (*P* < 0.05), which resulted as upregulated in ATH group.

The expression trends in CTRL and HF secretome samples of CHI3L1 and Cat-D were validated by Western blot, confirming the results obtained by mass spectrometry (Figure S1 in Supplementary Material).

### 3.3. Pathway Analysis and Tissue Immunohistochemistry

**Figure 3:**
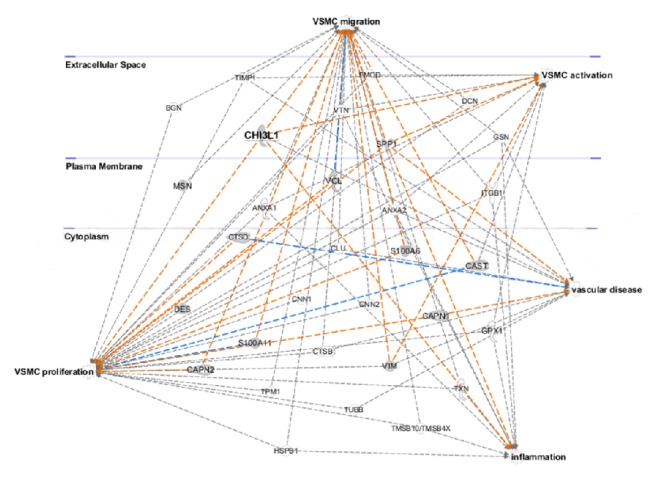
IPA of a subset of 31 proteins identified in the secretome of RCA specimens. Pathways were generated based on the information stored in IPA Knowledge base. Nodes correspond to the 31 proteins and are reported with their Gene Codes. Extracellular space, cytoplasm, and plasma membrane proteins are shown based on IPA classification. Relationships with disease and cellular functions are evidenced. The relationships that are predicted to lead to activation and to inhibition and those not predicted are reported in orange, blue, and grey, respectively.

IPA was used to highlight pathways and diseases which involve secreted proteins. The most represented pathways were those related to cell proliferation, migration, and VSMC activation. Among the proteins that are implicated in atherogenesis and vascular diseases, CHI3L1 was the only one that is predicted as linked to all pathways (Figure 3).

**Figure 4:**
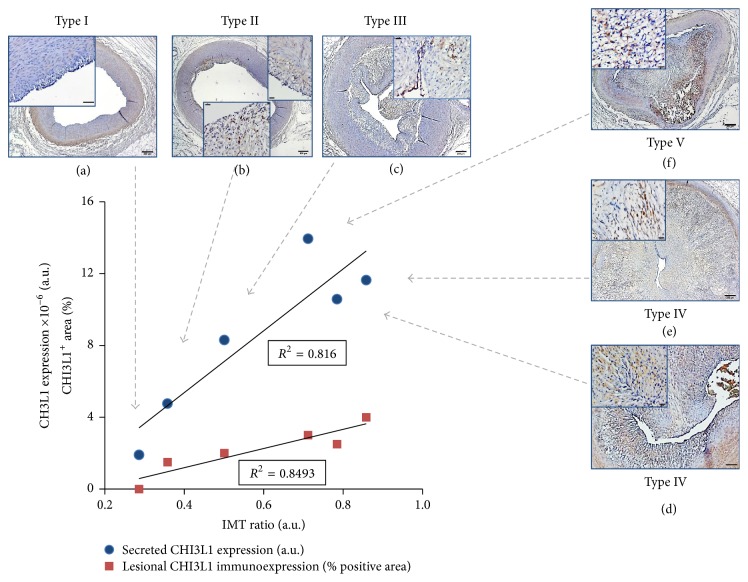
Correlation between secreted and tissue coronary CHI3L1 expression (by LC-MS/MS analysis and immunohistochemistry, resp.) and IMT ratio of corresponding segments at different Stary stages. Representative photomicrographs at low (bar = 200 *μ*m) and high magnification (enlargements of intimal lesion area, insets; bar = 50 *μ*m) from CHI3L1 antibody labeled cross sections of HF RCA segments (from (a) to (f)), according to ATS grade (Stary types I to V) demonstrate the correlation of CHI3L1 expression (dark brown staining indicates positivity) and of ATS stage with secreted CHI3L1 values (arrows). Secreted CHI3L1 expression (blue dots) and CHI3L1 immunoreactivity, expressed as percentage of intralesional positive cell area (red squares), hold direct and significantly linear relations (*R* ^2^ > 0.8) with the IMT ratio of the same segments.

On the basis of these results, anti-CHI3L1 antibody was used to quantify tissue expression of this protein and associate it with localization of other lesional VSMC markers and with ATS grade and morphometric indexes of lesion extent (Figure 4).

Arterial tissue immunoreactivity to anti-CHI3L1 antibody was quantified as percentage of intralesional positive-staining area in all RCA segments of CTRL and HF cases.

**Figure 5:**
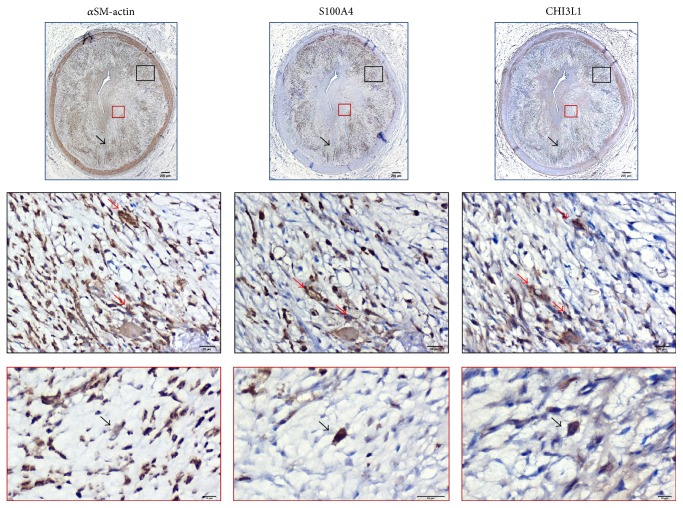
Representative photomicrographs of immunostained consecutive cross sections of RCA in a typical ATH case of HF group (immunopositive cells are dark brown). Immunostained sections demonstrate tissue codistribution (top row) and cell colocalization (middle and bottom rows) of anti-CHI3L1 with anti-*α*SM-actin and with anti-S100A4 antibodies in a fibrolipid plaque. Enlargements of plaque regions (black and red insets) are displayed in middle and bottom row, respectively. Plaque areas labelled by anti-*α*SM-actin, anti-S100A4, and anti-CHI3L1 antibodies are indicated (top panels, black arrows; bar = 200 *μ*m) and colocalization of all three antibodies in the same cell type evidenced (bottom panels, black arrows; bar = 10 *μ*m). CHI3L1 immunopositive cells resulted also in *α*SM-actin or S100A4 positive or both (middle panels, red arrows; bar = 20 *μ*m).

Anti-CHI3L1, anti-*α*SM-actin, and anti-S100A4 antibodies labelled the same region in all lesion types observed in adjacent consecutive HF RCA sections, demonstrating a consistent tissue codistribution. Colocalization of the three antibodies in the same cell type was also observed in several microscopic fields of HF RCA ATH cases (Figure 5).

ELISA for CHI3L1 detection was performed on end-diet plasma samples of the same CTRL and HF animals and the circulatory expression of the protein resulted as 2.6 ± 0.4 ng/mL in CTRL samples (mean ± SD) and 12.4 ± 3.5 ng/mL in HF samples (mean ± SD) (*P* value = 0.001).

## 4. Discussion

Different proteomics approaches have been used to search for biomarkers of ATS presence and severity. The majority of these studies in humans concern carotid (from surgery) and coronary arteries with overt disease and plaque complication features [24].

Body fluids, particularly blood, are the samples of choice for biomarker discovery in medicine since they can be easily and noninvasively collected. However, plasma proteomics profiling has turned out to be extremely challenging due to the wide dynamic range of proteins, the corresponding intrinsic low abundance of potential biomarkers and the huge individual heterogeneity of the samples. For these reasons, in the last years many researchers focused on the secretome analysis of cells and tissues with the expectation that identified putative biomarkers could be traced back in body fluids with more sensitive and targeted analyses [25].

Recently, we designed and assessed a gel- and label-free LC-MS/MS workflow that was used to produce a proteome profile concerning human atherosclerotic carotid plaque and secreted proteins from cultured cells [26, 27].

In the present work, the overall workflow was utilized to study the secretome-contained proteins of coronary arterial segments in order to characterize the early phases of plaque formation and growth, to identify molecular markers associated with pathology grade, and to evaluate their translation into the clinical field.

To this end, a swine model of coronary atherogenesis was used, and atherosclerotic changes of different stage, extent, and distribution along the three main coronaries were observed following high fat, cholesterol-enriched diet. Despite the overall variability of the lesion distribution in the coronary segments that we analyzed (Table S4 in Supplementary Material), a four-month high fat diet treatment was able to induce in all cases coronary atherosclerotic changes and at least one atheroma lesion. Conversely, no changes or only type I preatherosclerotic initial changes were observed in femoral arteries, a finding supported by previous studies of porcine coronary as compared to iliac/femoral artery atherosusceptibility during diet treatment [15, 28]. However, we cannot exclude the idea that a comprehensive histologic examination of all femoral segments, in addition to those sampled for the secretome analysis, could have evidenced more advanced atherosclerotic changes also in this artery.

LC-MS/MS analysis of the whole secretome content allowed identifying 224 proteins, among which 17 were differentially expressed between CTRL and HF cases.

*t*-test between HF FA and CTRL groups, both without significant atherosclerotic lesions, showed significant differential expression in glyceraldehyde 3-phosphate dehydrogenase, desmin, prelamin, glutathione peroxidase 1, and apolipoproteins A-I and A-IV, which was attributed to events induced by different diets: an atherogenic diet-dependent modulation of lipid and glucose metabolism-associated proteins and diet induction of free radicals modulating factors related to oxidative stress. Comparison between RCA segments of CTRL and HF groups showed that the most represented differentially expressed proteins were VSMC intracellular proteins and ECM factors related to VSMC activation and synthesis.

Most of the VSMC proteins (moesin, vimentin, and desmin) that resulted in upregulation in RCA HF atherosclerotic samples, but not in HF FA samples, are related to the adhesion pathway and are strongly modulated during VSMC phenotype switch and involved in VSMC capacity to migrate [29]. This was confirmed also by bioinformatics pathway analysis (IPA software), which supported the involvement of these proteins in vascular disease and in VSMC proliferation/migration. Additionally, a relationship was found between their expression and the lesion stage and extent.

Furthermore, a significant modulation of ECM components, mostly synthesized by synthetic VSMCs, such as Biglycan, Hyaluronan, and Osteopontin, was observed in the secretome samples of HF RCA but not in HF FA samples. Proteoglycans are reported to bind cytokines and growth factors causing inflammation during ATS development [30] and may be involved in lipid retention, thus contributing to the early phases of lesion formation [31, 32]. Also many apolipoproteins (apolipoproteins A-I, A-IV, and E) are modulated in our model supporting this hypothesis.

CHI3L1 was the most relevant upregulated protein identified in HF RCA samples, which was (i) differentially expressed between CTRL, pre-ATH, and ATH samples, (ii) strongly associated with plaque size/extent and Stary stage, and (iii) immunohistologically related to a prevalent VSMC plaque composition. The site-specific association with lesion stage and size was validated for the full spectrum of diet induced lesions in samples used for secretome analysis and immunohistochemical characterization, enabled to establish the tissue and cell colocalization of CHI3L1 immunoexpression with *α*SM-actin/S100A4 positive-staining areas and migratory VSMC phenotype.

At variance with previous human studies [33, 34], we did not observe the maximal anti-CHI3L1 immunoexpression in macrophages and lipid laden macrophages (Figures S2 and S3 in Supplementary Material). Conversely, maximal CHI3L1 expression was found in *α*SM-actin/S100A4 positive cells, supporting its prevalent synthesis in the activated VSMC phenotype within developing atherosclerotic lesions. This difference could be explained by the more advanced stage and different cell composition of the complicated human carotid plaques as compared to coronary plaques from our experimental model of atherogenesis.

It is reported that the expression of CHI3L1 is enhanced in aorta of patients with coronary atherosclerosis and is significantly correlated with atherosclerotic risk factors [35], although increased expression of CHI3L1 in human atherosclerotic lesions is primarily associated with production and activation of inflammatory factors [36]. Despite being associated with VSMC presence in atherosclerotic plaque [37] and with VSMC activation towards a synthetic phenotype [38], this protein has never been hitherto shown to be directly correlated to coronary ATS grade and extent during atheroma formation.

CHI3L1 expression was also measured by ELISA in plasma samples of pigs fed on standard and high fat diet. The statistically significant upregulation of its circulatory expression consolidates the association between the expression of this marker and the presence of CAD in this experimental model of atherogenesis and supports its amenability to be used as marker of ATS severity in the clinical context.

Altogether, these data confirm that VSMC activation towards a migratory/synthetic phenotype may play a pivotal role in the early formation and progression of coronary atheromas and suggest that VSMC-derived/-secreted molecules can be usefully exploited as plaque stage/size related biomarkers in CAD.

### 4.1. Study Limitations

The major drawbacks in atherogenesis research are (i) difficulties in investigating the early phase of the pathology in the clinical context and (ii) discrepancies between experimental models and human characteristics of the disease, which can limit the relevance of results. The use of highly unbalanced diets and/or genetic manipulation are the only possible choices to produce appreciable atherosclerotic changes within few months as technically required. The atherogenic diet adopted in our study to induce accelerated coronary lesions in pigs could make it difficult to extrapolate results credibly from the experimental to the clinical context; however, the diet-independent relation to site-specific and feature-specific characteristics of coronary atherosclerotic plaque suggests our proteomics findings as realistically informative in the field of marker discovery for disease initiation and evolution.

This hypothesis is supported by the evidence that, despite intragroup variability of lesion grade and distribution, morphometric indexes of single RCA segments processed for secretome analysis are directly and significantly related to the average coronary values of the corresponding cases (see Figure S4 and Table S4 in Supplementary Material), thus extending the relevance of site-specific proteomics results to the entire atherosclerotic coronary tree.

Despite the limited number of samples analysed, the observation that several plaque-secreted proteins identified in our animal model have been previously found overexpressed also in human atherosclerotic tissue and blood [35, 39] indirectly reinforces the translational utility of our study.

## 5. Conclusion

The outcomes of this study are the following:

1. The most relevant VSMC-secreted proteins are related to coronary ATS stage and extent, suggesting that VSMCs regulate and contribute to the initial stages of CAD. Moreover, profiling data indicate a general reorganization of VSMC-based pathways connected with CAD severity, supporting the role of activated VSMCs in the evolution of coronary lesions from fatty streak to atheroma.
2. CHI3L1 is the most strongly associated protein with coronary ATS grade and extent.
3. Immunohistochemistry suggests prevalent localization of CHI3L1 in S100A4 positive VSMCs and supports the stage-specific distribution of this marker in the full spectrum of coronary lesions, from initial fatty streak to fibroatheroma.
4. ELISA of CHI3L1 confirms a statistical significant association between its circulatory level and the presence of CAD.

This approach can help identify circulating markers of early plaque development and/or fast growth that could be validated in a large clinically characterized cohort of patients, contributing to improving risk assessment of primary coronary events in CAD.

## Supplementary Material

Table S1: List of identified proteins in secretome of HF and CTRL artery samples.
Table S2: List of identified peptides for protein in secretome of HF and CTRL artery samples
Table S3: Protein list and MS-based protein expressions
Table S4, page 1. Histomorphometric data of 134 coronary segments analysed in HF group.
Figure S1, page 2. Box plots of Western blot results of CHI3L1 and Cat-D in secretome samples.
Figure S2, page 3. Immunostaining micrographs of cell co-localization of anti CHI3L1 with anti CD107a and with anti Mac387
Figure S3, page 4. Double immunostaining micrographs of anti-CHI3L1 and anti-CD107a.
Figure S4, page 5. Scatter plot of the relation between average IT values of RCA secretome samples and average IT of all other coronary segments analyzed in each HF case.
Figure S5, page 6. Plot of Principal Component Analysis of MS data of secretome samples.

## Figures and Tables

**Table 1:** Histomorphometric results of RCA and FA segments in 6 CTRL and 6 HF cases.

	CTRL RCA (6)	HF FA (6)	HF RCA (6)	RCA pre-ATH (3)	RCA ATH (3)
*N*/*Ntot. Stary types I*–*III*	*0/6*	*2/6*	*3/6*	**3/3**	**0/3**
*N*/*Ntot. Stary types IV-V*	*0/6*	*0/6*	*3/6*	**0/3**	**3/3**
IT (mm)	0.03 ± 0.02	0.06 ± 0.05	0.50 ± 0.44	**0.13 ± 0.05**	**0.86 ± 0.36**
IMT ratio (a.u.)	0.11 ± 0.04	0.15 ± 0.05	0.58 ± 0.24	**0.32 ± 0.11**	**0.77 ± 0.07**
LA (mm^2^)	0.04 ± 0.02	0.06 ± 0.04	2.61 ± 3.42	**0.30 ± 0.27**	**5.00 ± 3.64**
LA ratio (a.u.)	0.03 ± 0.02	0.05 ± 0.02	0.97 ± 1.13	**0.14 ± 0.10**	**1.75 ± 1.10**

Number of segments (*N*) and average value ± SD of maximal IT (IT), maximal intima to media thickness ratio (IMT ratio), lesion area (LA), and lesion to intact wall area ratio of FA and RCA in CTRL and HF cases are reported. RCA segments showing Stary types I–III (RCA pre-ATH) and those displaying Stary types IV-V lesions (RCA ATH) are also grouped separately.

**Table 2:** Differential protein expression between HF RCA and CTRL cases.

Localization	Gene name	Protein name	Coronary HF/CTRL	*P* value	SecretomeP	Plasma	Role in atherogenesis
Cellular	G3P	Glyceraldehyde-3-phosphate dehydrogenase	Up	0.05	*√*	*√*	Glucose metabolism
	CATD	Cathepsin D	Up	0.009	*√*	*√*	Macrophage phagocytosis, lipid efflux [40, 41]
	DESM	Desmin	Up	0.04	*√*		Focal adhesion [29]
	VIME	Vimentin	Up	0.02	*√*		Focal adhesion [29]
	CPNS1	Calpain	Up	0.05	*√*		Calcium binding, proatherogenic [42]
	ICAL	Calpastatin	Up	0.05	Not predicted		Calcium binding, calpain inhibitor
	MOES	Moesin	Up	0.005	*√*		Focal adhesion [43]
	CH3L1	Chitinase-3-like protein 1	Up	0.004	*√*	*√*	Adhesion and migration [37]
	S10A6	Protein S100-A6	Up	0.05	*√*	*√*	RAGE ligand and marker of MI [44]
	LMNA	Prelamin-A/C	Up	0.03	Not predicted		Oxidative stress, vascular aging [45]
	GPX1	Glutathione peroxidase 1	Up	0.05	*√*		Oxidative stress

Extracellular matrix	PGS1	Biglycan	Up	0.04	*√*	*√*	Lipoprotein retention [46]
	HPLN1	Hyaluronan and proteoglycan link protein 1	Down	0.000033	*√*	*√*	Plaque stability, atheroprotection [47]
	OSTP	Osteopontin	Up	0.04	*√*	*√*	Secondary carotid events marker [39]
	APOA1	Apolipoprotein A-I	Up	0.01	*√*	*√*	HDL constituent, atheroprotection [48]
	APOE	Apolipoprotein E	Up	0.0007	*√*	*√*	Atheroprotection [49]
	APOA4	Apolipoprotein A-IV	Down	0.03	*√*	*√*	Antioxidative, atheroprotection [50]

First column: biological localization of proteins. Second column: gene name. Third column: protein names reported according to SwissProt 2013 database. Sixth column: secretion potential as predicted according to SecretomeP software. Seventh column: presence in plasma, according to Human Protein Reference database (http://www.hprd.org). Eighth column: roles in atherogenesis, as suggested in the literature.
