# Supplementary material for: Site-Specific Secretome Map Evidences VSMC-Related Markers of Coronary Atherosclerosis Grade and Extent in the Hypercholesterolemic Swine
Source: Dis Markers. 2015 Aug 25;2015:465242. doi: 10.1155/2015/465242 (PMC4561865; doi:10.1155/2015/465242)
Supplement: Supplementary file 1 — Table S1: List of identified proteins in secretome of HF and CTRL artery samples. Table S2: List of identified peptides for protein in secretome of HF and CTRL artery samples Table S3: Protein list and MS-based protein expressions Table S4, page 1. Histomorphometric data of 134 coronary segments analysed in HF group. Figure S1, page 2. Box plots of Western blot results of CHI3L1 and Cat-D in secretome samples. Figure S2, page 3. Immunostaining micrographs of cell co-localization of anti CHI3L1 with anti CD107a and with anti Mac387 Figure S3, page 4. Double immunostaining micrographs of anti-CHI3L1 and anti-CD107a. Figure S4, page 5. Scatter plot of the relation between average IT values of RCA secretome samples and average IT of all other coronary segments analyzed in each HF case. Figure S5, page 6. Plot of Principal Component Analysis of MS data of secretome samples. [file 465242.f1.zip › 465242.f1/465242.f1.docx]

**Table S4: Histomorphometry data of 134 coronary segments analysed in HF group cases.**

|  | **Intact**  **(n= 21)** | **Type I (n=23)** | **Type II (n=31)** | **Type III (n=17)** | **Type IV**  **(n=20)** | **Type V**  **(n=22)** |
| --- | --- | --- | --- | --- | --- | --- |
| **IT (mm)** | 0.02 ± 0.02 | 0.10 ± 0.06 | 0.25 ± 0.11 | 0.56 ± 0.16 | 1.07 ± 0.46 | 1.52 ± 0.48 |
| **IMT ratio (a.u.)** | 0.15 ± 0.07 | 0.44 ± 0.21 | 0.65 ± 0.39 | 1.69 ± 0.56 | 3.87 ± 2.13 | 6.53 ± 2.17 |
| **LA (mm^2^)** | 0.01 ± 0.01 | 0.26 ± 0.16 | 0.58 ± 0.40 | 1.12 ± 0.45 | 2.94 ± 2.00 | 5.32 ± 2.32 |
| **LA ratio (a.u.)** | 0.01 ± 0.01 | 0.07 ± 0.05 | 0.16± 0.11 | 0.37± 0.11 | 0.54± 0.16 | 0.73± 0.12 |

134 segments harvested from the 3 main coronary arteries in HF group are subdivided in intact and atherosclerotic according to Stary histological classification for human CAD. Average values of cross-sectional intimal thickness (IT), intima to media thickness (IMT) ratio, lesion area (LA) and LA to intact wall area ratio (LA ratio) for each class of segments are reported. Individual values are calculated as the average of values assessed in 5 serial cross sections at maximal lesion extent.


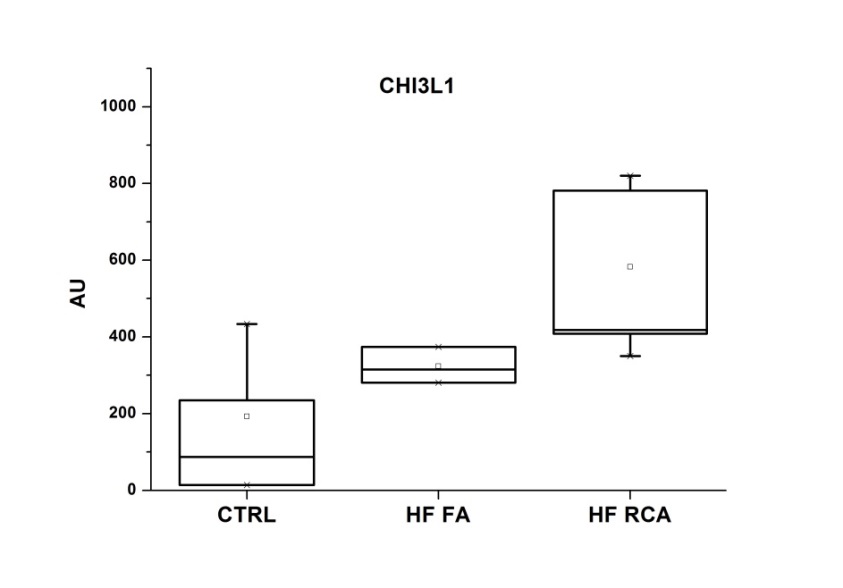

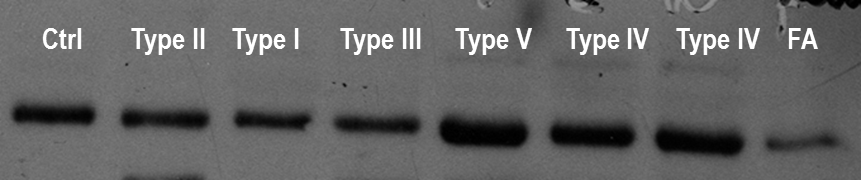


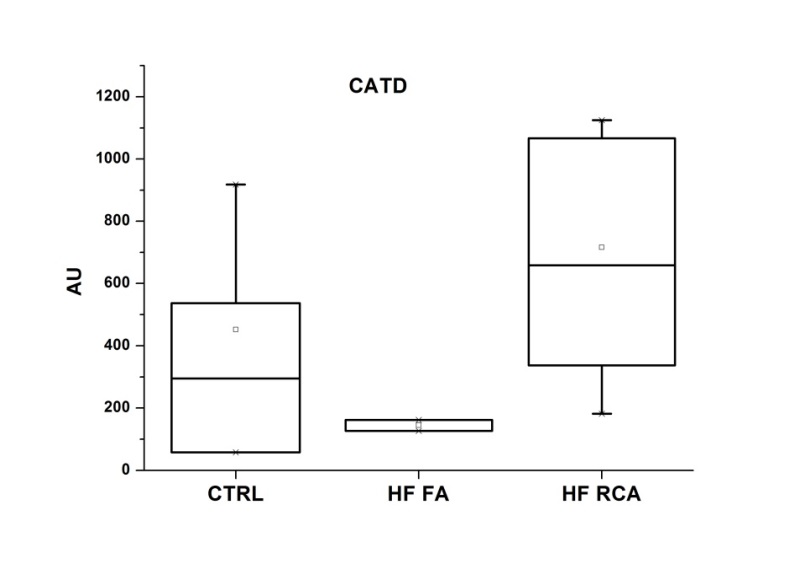
***
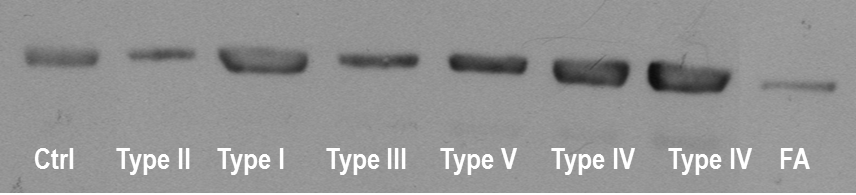
***

***Figure S1:*** *Box plots of Western blot results of CHI3L1 and Cat-D in secretome samples. CTRL (N=6), HF FA (N=6) and HF RCA (N=6). CHI3L1 resulted in CTRL 192±185 counts, in HF FA 323±47 counts and in HF RCA 583±212 counts (mean ± SD). Cat-D resulted in CTRL 452±367 counts, in HF FA 145± 25 counts and in HF RCA 716±391 counts (mean ± SD).*

*
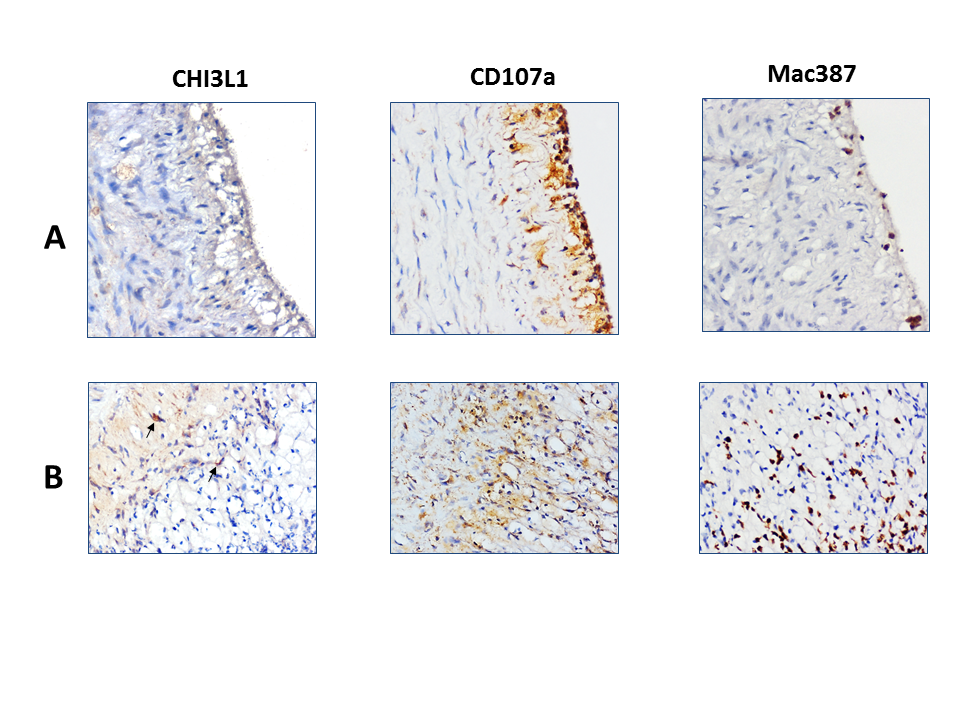
*

***Figure S2.*** *Immunostaining of consecutive cross sections demonstrates no cell co-localization of anti CHI3L1 with anti CD107a and with anti Mac387 antibodies in an initial fatty streak type I (A) and in a preatheroma type III lesion (B) of a HF segment. Anti-CD107a (mouse, clone PG-M1, Thermo Scientific, diluted 1:150) binds lysosome LAMP-1 membrane glycoprotein, a porcine myelomonocytic (Developmental and Comparative Immunology 33 (2009) 284–298 Porcine myelomonocytic markers and cell populations. A. Ezquerra, C. Revilla, B. Alvarez, C. Perez, F. Alonso, J. Domınguez ) and macrophage foam cells marker (Jerome, W. G., Cox, B. E., Griffin, E. E. & Ullery, J. C. Lysosomal cholesterol accumulation inhibits subsequent hydrolysis of lipoprotein cholesteryl ester. Microsc. Microanal. 14, 138–49 (2008)). Anti-MAC387 (S100A8/A9, calprotectin clone MAC387 Thermoscientific, diluted 1:1000) is a classic macrophage marker (N. Solanes, M. Rigol, J. Ramırez et al. Histological Basis of the Porcine Femoral Artery for Vascular Research Anat. Histol. Embryol. 34, 105–111 (2005))CD107a and MAC387 positive cells are distinct from CHI3L1 positive cells (B, black arrows), suggesting no significant expression of CHI3L1 in macrophages - foam cells and in macrophages respectively. 40x original magnification, bar = 20µm.*

*
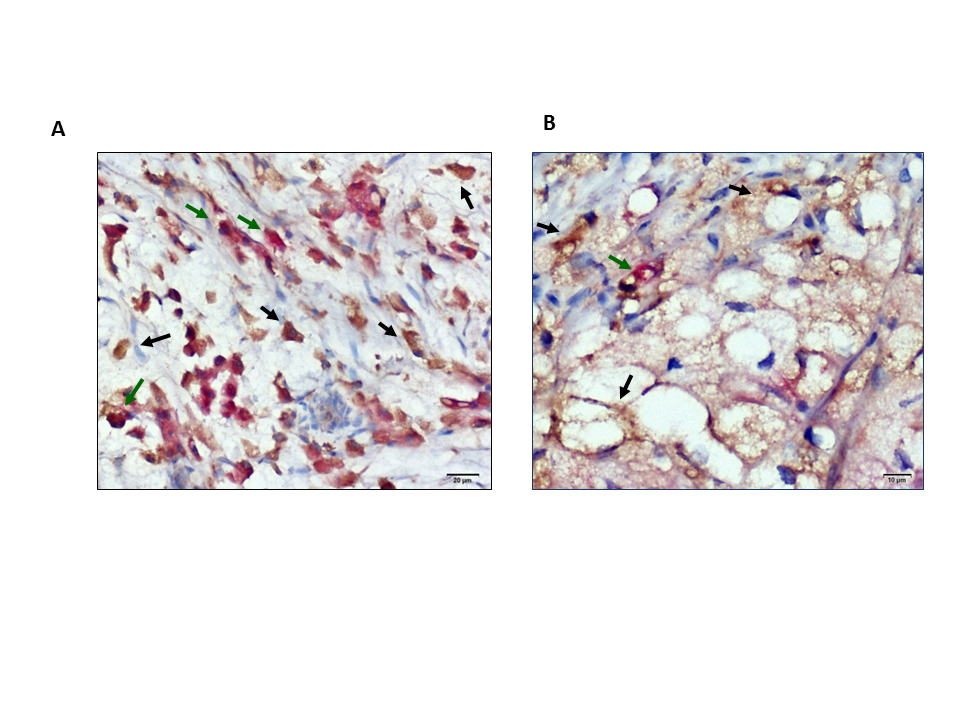
*

***Figure S3.*** *Micrographs of a fibrolipid plaque (Stary type V) of a HF RCA ATH segment showing double immunostaining of anti-CHI3L1 and anti-CD107a antibodies, performed using Vectastain Elite ABC reagent incubation in phosphatase (AP) substrate solution and Permared-AP stain for CHI3L1 and DAKO LSAB system HRP and subsequent peroxidase substrate solution (DAB) incubation for CD107a. CD107a positive (brown) cells are distinct from CHI3L1 positive (red) cells (black and green arrows respectively), suggesting low/no immunoexpression of CHI3L1 in macrophages-foam cells. 40x original magnification, bar = 20µm and 10µm in A and B respectively.*


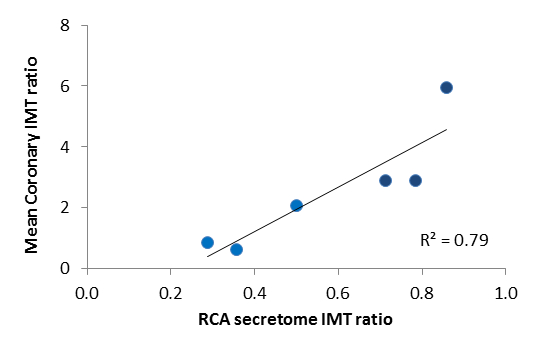


***Figure S4:*** *Linear relation between average IT values of HF RCA pre-ATH (light blue dots) and HF RCA ATH (dark blue dots) secretome segments of each HF case and the corresponding average values of IT of all other coronary segments sampled and analyzed in each HF case (R^2^ = 0,72, P<0,05), supporting that coronary lesion features of single RCA secretome segments are representative of the entire coronary arterial tree in each HF case.*

*
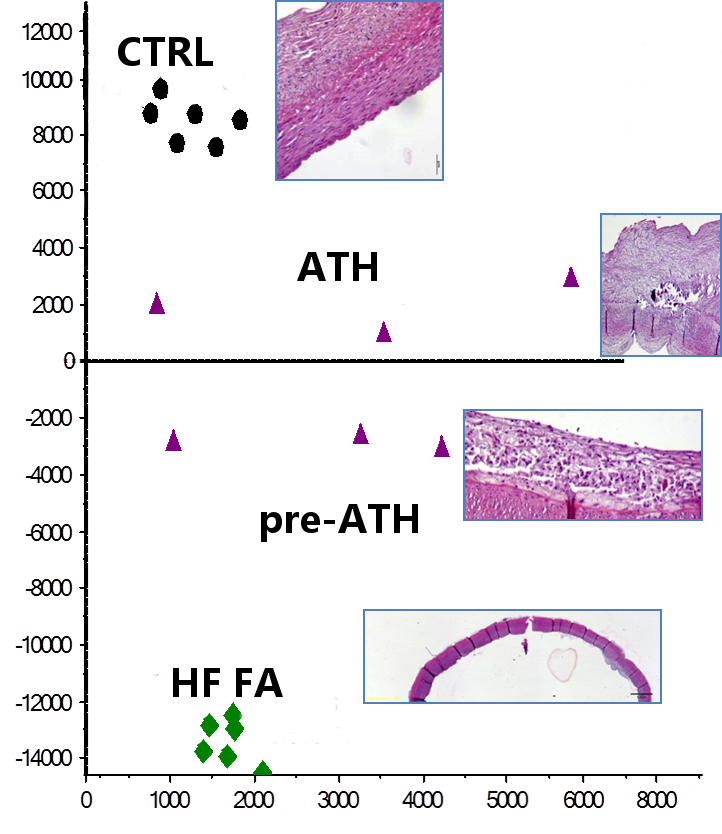
*

***Figure S5:*** *Principal Component Analysis of MS data of secretome samples (CTRL N=6, HF FA N=6 and HF RCA N=6 separated as ATH and pre-ATH). X axis: PC1 and Y axis: PC2*
